# Development of a Genomic Resource and Identification of Nucleotide Diversity of Yellow Perch by RAD Sequencing

**DOI:** 10.3389/fgene.2019.00992

**Published:** 2019-10-14

**Authors:** Liang Guo, Hong Yao, Brian Shepherd, Osvaldo J. Sepulveda-Villet, Dian-Chang Zhang, Han-Ping Wang

**Affiliations:** ^1^Aquatic Genetics and Breeding Laboratory, Ohio State University South Centers, Piketon, OH, United States; ^2^Key Laboratory of South China Sea Fishery Resources Exploitation and Utilization, Ministry of Agriculture and Rural Affairs, South China Sea Fisheries Research Institutes, Chinese Academy of Fishery Sciences, Guangzhou, China; ^3^USDA-ARS-School of Freshwater Sciences, University of Wisconsin-Milwaukee, Milwaukee, WI, United States; ^4^School of Freshwater Sciences, University of Wisconsin-Milwaukee, Milwaukee, WI, United States

**Keywords:** RAD-seq, yellow perch, polymorphic SSR, germplasm collection, genotyping, aquaculture, conservation

## Introduction

Yellow perch, *Perca favescens*, is a freshwater fish, natively distributed in temperate and subarctic areas of North America, and its abundance and native distribution center are in the lower Great Lakes region (Craig, 1987; Sepulveda-Villet et al., 2009). Its long-term population distribution has been shaped by global climate change, mainly by Pleistocene glaciations and geophysical modifications (Sepulveda Villet and Stepien, 2012), with short-term population dynamics influenced by factors such as adaptive competition and capture fisheries (Coots, 1956; Malison, 2003; Marsden and Robillard, 2004; Houde et al., 2014; Bodamer Scarbro, 2014). During the Pleistocene glaciations, populations persisted in the three primary North American glacial refugia: Missourian, Mississippian, and Atlantic. Current yellow perch populations are attributed to at least two primary glacial refugia and divided into six major geographic regions: Northwest Lake Plains, Great Lakes watershed, Lake Champlain, US North Atlantic coastal, South Atlantic coastal, and Gulf coastal (Sepulveda Villet and Stepien, 2012). This species is in high demand for human consumption in the Great Lakes Region and a high-priority species for aquaculture production (Malison, 2003). The production of the species, however, largely depends on capture fisheries in the United States and Canada, principally the Great Lakes. While the demand for this species is approximately 5 kilotons each year, its production in aquaculture is only 100 tons each year, according the record of food and agriculture organization of the United Nations (Malison, 2003; FAO, 2018). In addition, wild harvest drastically declined from the peak harvest in the 1950s and 1960s, and even more so during the 1980s and 1990s (Malison, 2003). All these factors, including the large population fluctuation, sharp capture production decline, and high demand in aquaculture, put pressure on the basic need for genetic research, broodstock management and resource conservation.

Previous studies utilized allozymes, mitochondrial DNA, and single-sequence repeats (SSRs) as genetic markers to characterize population genetic structure (Leclerc et al., 2000; Brown et al., 2007). Restriction site-associated DNA sequencing (RAD-Seq) has emerged as a powerful technique for high-throughput single-nucleotide polymorphism (SNP) discovery and genotyping (Baird et al., 2008). For paired-end RAD reads assemblies, usually the forward reads are first clustered, whether the data are from an individual (Wang et al., 2016) or population (Hohenlohe et al., 2013), and then the reverse reads within the same cluster are assembled according to the paired-end relationships.

SSRs have been widely used in fisheries for resource investigation and management and in aquaculture for strain identification, parentage assignment, genetic linkage map construction, and quantitative trait loci mapping (Sundaray et al., 2016). In the traditional approach of SSR development, the repeat sequences were first enriched by hybridization with biotinylated oligonucleotides and then sequenced (Chistiakov et al., 2005). With more genome and EST sequences released with the aid of Sanger sequencing and next generation sequencing (NGS) technology, SSR motifs can be searched in sequence databases (Zhan et al., 2009). However, converting these motifs to SSR markers still needs validation for polymorphism and actual polymerase chain reaction (PCR) amplification. Fortunately, SSRs still catch attention and could be directly genotyped in a sequenced population (Tang et al., 2008; Cardoso et al., 2013; Willems et al., 2014; Cantarella and Agostino, 2015; Vukosavljev et al., 2015). Furthermore, another important reason to focus on SSRs is to expand our capacity to understand SSR evolution and their influence on traits (Willems et al., 2014).

Herein, we applied RAD-Seq data for polymorphic SSR development, with the aim of showing how these sequences and markers would benefit the yellow perch conservation and aquaculture genomics research. First, we combined the advantage of longer sequence length in MiSeq platform and higher throughput of the HiSeq platform to assemble the RAD-Seq contigs. Then, a large amount of SNPs and SSRs were genotyped. Third, nucleotide diversity was assessed using developed SNPs. Fourth, a random subset of newly discovered SSRs was validated by PCR.

## Data

**Figure 1:**
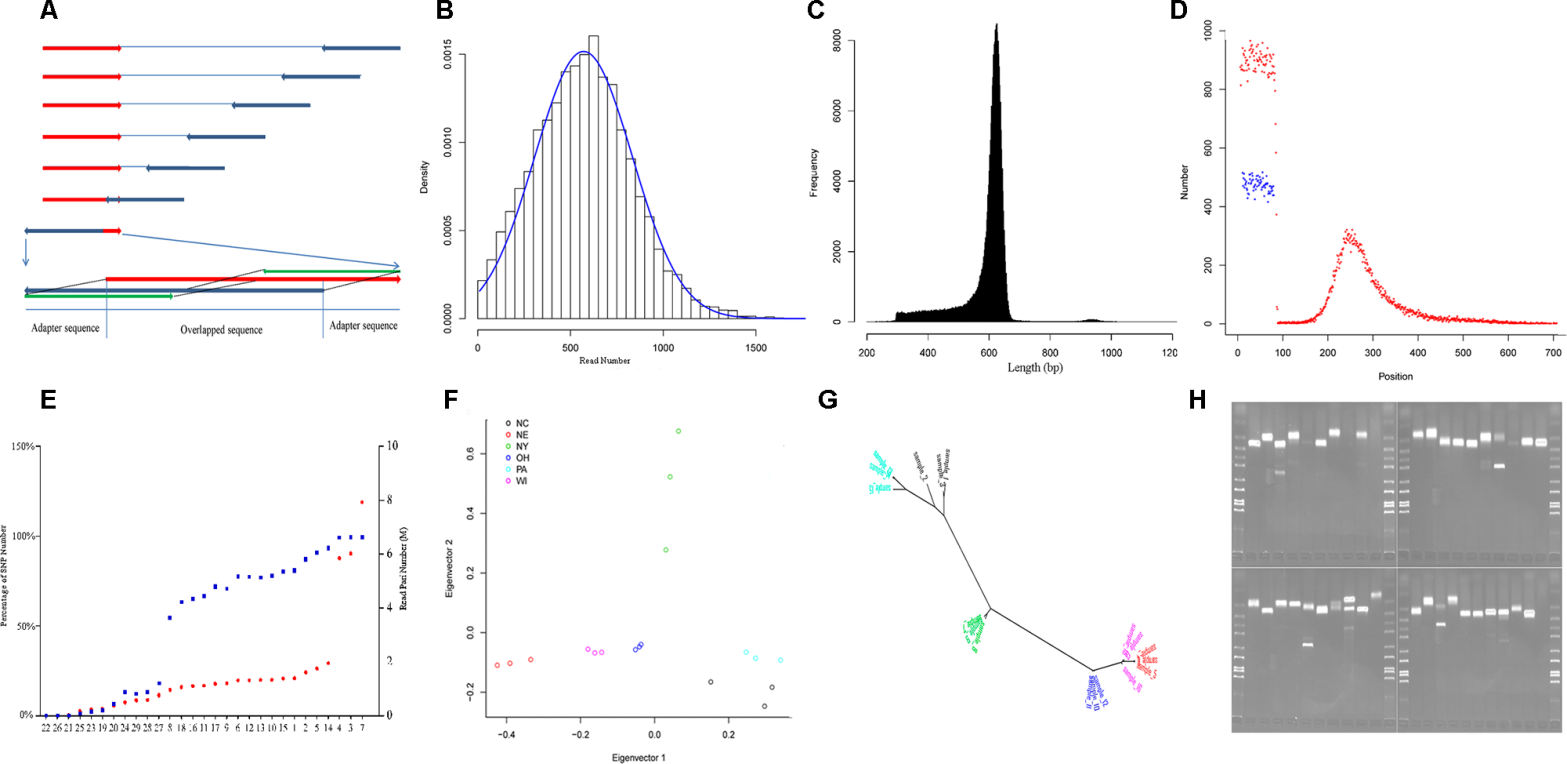
Characteristics of yellow perch RAD-Seq. **(A)** Illustrating the removal of adaptor sequences at the 3′ end of read. The red arrows show the forward reads, and blue arrows show the reverse reads. Usually, each read contains adaptor sequence at the 5- end and removed routinely. The adaptor sequence would appear in 3′ end when the read length is longer than the library size. Based on the characters of rare Indel and overlapping genome sequence fragment, the 5′ end sequence fragment probe (green) in the forward read (red) was used to scan the reverse read (dark blue) in the direction from 3′ end to 5′ end with a step size of 1 bp. Once the distance fell below a certain threshold value, the sequence fragment located on the 3′ end of the matched sequence fragment was treated as an adapter and then removed. The scan was also applied to the forward read. **(B)** The read number that clustered for each RAD-tag. The blue line shows the expected distribution fitting Gaussian distribution. **(C)** The distribution of final contig length. **(D)** The distribution of SNPs along the contig (red points: SNPs in the first dataset, blue points: SNPs in the second dataset). **(E)** The percentage of SNP number for each individual in the whole SNPs (blue points, left axis) in the first dataset and read pair number for each individual after removing duplication (red points, red axis). The nine individuals with less than 50% of total SNPs were removed in population genetics. **(F)** The distribution of the 18 individuals from six strains (**Table 1**) along the first and second principal components. **(G)** The phylogenetic tree for the same individuals in graph G with the same color schedule. **(H)** The electrophoretogram for the 40 randomly chosen SSRs. The last second lane on the right-down figure is negative control without primer inside.

We applied RAD-Seq to yellow perch geographic demes to develop large numbers of polymorphic genetic markers, including SNPs and SSRs, and to evaluate nucleotide diversity of this species. We achieved 179.9 M read pairs in total and 6.3 M in average on HiSeq platform, and 2.4 M read pairs and 0.8 M in average on MiSeq platform. The average coverage was 11.2 fold in forward reads. In average, 1.0% and 37.6% reads from HiSeq and MiSeq platforms contained adaptor sequences at the 3′ end. In total, reads allocated into 351,578 RAD-tags were selected to assemble contigs, and 258,056 pairs of forward and reverse contigs were merged to final contigs, in which 56,845 (22%) contained the separator of 10 “N.” The length of the final contigs was 605 ± 71 bp (mean ± SD, **Figure 1C**), and the total length of all final contigs summed was approximately 152 Mbp, which accounts for 16.9% of the genome sequence length (*C* value: 0.92; Peterson et al., 1994). The lengths of the contigs assembled from both Miseq and HiSeq reads (617 ± 63 bp, mean ± SD) were longer than those only from HiSeq reads (546 ± 96 bp, mean ± SD) (*p* < 0.001, two-sample Wilcoxon tests); 40.3%, 19.1%, 18.2%, and 3.1% contigs mapped to the genomes of European seabass, Nile tilapia, three-spined stickleback, and zebrafish, respectively, in which the rank was consistent with the expected order of taxon.

Variants were detected, and three variant datasets were used for primer design, genetic diversity estimation, and population structure inference, respectively. The first and second datasets contained 41,736 and 33,186 SNPs, respectively. They were both uniformly located on the forward contig (**Figure 1D**). There were no SNPs located on enzyme recognition sites, or any inflation at end of the forward contig. The second dataset from 18 individuals was used to estimate genetic diversity, and each individual contained at least 50% of the total SNPs (**Figure 1E**). The total site number was 4,442,464, and the total nucleotide diversity was estimated as 0.00304 with 95% confidence intervals from 0.00303 to 0.00304. The third dataset contained 27,868 SNPs and was used to inference population structure. A principal component analysis was performed, and only the first component was significant (*p* = 0.027), which explains 45.8% of the total variance (**Figure 1F**). The principal component analysis distinguished the origin and distribution of the strains examined, wherein the NY, PA, and NC1 strains were inferred to originate from the Atlantic refugium, and the NE populations were inferred to originate from the Missourian refugium. The NY strains were distributed in the North Atlantic region, while the PA and NC1 strains belonged to the South Atlantic coastal region (**Table 1**). The first principal component reflected the migration origin of the strains. The second principal component separated the divergence along the Atlantic coastal region. The phylogenetic tree also showed the main divergence of origin of different strains (**Figure 1G**).

**Table 1:** Description of samples and statistic of reads.

Sample code	Index	Strain	Platform	Read pair (M)		Second dataset^2^	Distribution
				**Raw**	**Effective** **^1^**		
1	CCAAC	NC1	HiSeq	7.1	1.39	Y	South Atlantic coast
2	GAGAT	NC1	HiSeq	8.1	1.61	Y	
3	CGACGATACTTG	NC1	HiSeq	18.8	6.02	Y	
4	TCTGAGCGTACA	NE	HiSeq	16.0	5.85	Y	Northwest Lake Plains
5	GATCG	NE	HiSeq	8.4	1.76	Y	
6	GCATT	NE	HiSeq	6.3	1.31	Y	
7	ATGTGTCGCCAA	NY	HiSeq	25.6	7.93	Y	Lake Ontario
8	AAGGG	NY	HiSeq	4.4	0.96	Y	
9	ACACG	NY	HiSeq	6.1	1.20	Y	
10	CACAG	OH	HiSeq	7.1	1.33	Y	Lake Erie West
11	CAGTC	OH	HiSeq	6.0	1.11	Y	
12	CATGA	OH	HiSeq	6.2	1.31	Y	
13	TAGCA	PA	HiSeq	6.0	1.33	Y	Lake Erie East
14	TATAC	PA	HiSeq	9.2	1.95	Y	
15	TCAGA	PA	HiSeq	5.6	1.38	Y	
16	GACTA	WI	HiSeq	5.4	1.20	Y	Lake Michigan
17	AAAAA	WI	HiSeq	5.0	1.19	Y	
18	AACCC	WI	HiSeq	2.1	1.06	Y	North Atlantic coast
19	TATAC	MD	HiSeq	4.9	0.26	N	
20	TCAGA	MD	HiSeq	5.3	0.37	N	
21	CTTCCGG	MD	HiSeq	1.9	0.02	N	
22	TGGTATG	MD	HiSeq	1.0	0.01	N	South Atlantic coast
23	ATGTGTCGCCAA	NC2	HiSeq	2.8	0.24	N	
24	TCTGAGCGTACA	NC2	HiSeq	5.7	0.49	N	
25	TAGCA	NC2	HiSeq	3.3	0.17	N	
26	CGCACTC	NC2	HiSeq	1.6	0.02	N	
27	ATGTGTCGCCAA	NY	MiSeq	0.9	0.77	N	Lake Ontario
28	TCTGAGCGTACA	NE	MiSeq	0.7	0.58	N	Northwest Lake Plains
29	CGACGATACTTG	NC1	MiSeq	0.7	0.57	N	South Atlantic coast

^1^This column shows the number of read pairs after removing of duplication.

^2^Y indicates the individual was included in the second dataset, otherwise represented by “N”.

Among the total 255,305 contigs (length <800 bp), 42,752 (16%) contained 59,766 perfect SSRs, including 49,052 (82.1%), 6,299 (10.5%), 3,960 (6.6%), 314 (0.5%), and 141 (0.2%) of di-, tri-, tetra-, penta-, and hexa-nucleotide repeat motifs, respectively. These repeat motifs classified into four dimeric, 10 trimeric, 30 tetrameric, 50 pentameric, and 44 hexameric categories. The most common motifs of di-, tri-, tetra-, penta-, and hexa-nucleotide repeats consisted of AC/GT (68.5%), AAT/ATT (38.5%), AGAT/ATCT (23.6%), AGAGG/CCTCT (19.4%), and ACACGC/CGTGTG (36.9%) motifs. When considering imperfect SSRs, then the total number of SSRs increased to 73,703, and the most common repeats were changed to be AC/GT, AAT/ATT, AAAT/ATTT, AATTC/AATTG, and AACCCT/AGGGTT motifs. We took allele number as a measurement to evaluate the polymorphism of each type of motif. A total of 10,412 SSRs were then detected with at least two alleles. As with other studies in humans (Willems et al., 2014), the number of alleles is inversely correlated with motif length (*p* < 0.001, Kruskal–Wallis test) and positively correlated with length of alleles (Pearson correlation coefficient: 0.23, *p* < 0.001). To explore the genomic resource in yellow perch, primers were successfully designed for 3,830 SSRs with at least three alleles and flanked with sequence at least 200 bp at each side. The randomly selected 40 pairs of primers were validated using PCR, and 34 (85%) pairs showed expected bands, in which three pairs showed extra tidy bands outside the expected range (**Figure 1H**). The high validation ratio showed the assembled contigs were reliable, and the designed primers could be directly used in genotyping.

## Materials and Methods

### Sample Collection

Eight strains, NC1 (Perquimans River, 2010), NC2 (Perquimans River, 2006), NE (Sandhill lakes), NY (Erie Canal), MD (Choptank River), OH (Lake Erie), PA, and WI (Green Bay), were selected with three to four individuals sampled from each strain (**Table 1**). These samples captured the mainly native distribution region of this species. Genomic DNA was extracted from fin tissues using the method described by Li et al. (2007). All the samples were sequenced using paired-end RAD-Seq (Baird et al., 2008), in which three individuals were sequenced on MiSeq platform with 2 × 300 bp and others on HiSeq 2000 platform with 2 × 100 bp. The restriction enzyme was EcoRI, and the library size was approximately 600 bp.

### Contig Assembly

Reads were filtered and clustered using software Stacks version 1.42 (Catchen et al., 2013). The raw reads were filtered and separated using program process_radtags with default parameters without rescue of barcodes. Then, the forward reads were cut to be 85 bp in length. The reads from each individual were clustered using programs denovo_map.pl, rxstacks, cstacks, and sstacks with parameters of minimal depth for each stack, maximal mismatch allowed between stacks, and number of mismatches allowed between sample loci to be 3, 2, and 3. The highly repetitive catalogued loci were removed.

The forward and reverse reads that allocated into each RAD-tag were assembled separately, and the forward contig and reverse contig were merged into final contig. Before being allocated, the read pairs, without cutting in length, were processed using Hamming distance to filter sequence fragments that were actually adapter sequences in the 3′ end (**Figure 1A**). This step in trimming the adapter sequence was performed in the Perl script, trim_adaptorseq.pl, with sizes of probe 50bp and maximal distance threshold 5. Then the reads belonging to each RAD-tag (**Figure 1B**) were allocated using sort_read_pairs.pl and assembled separately with software CAP3 (Huang and Madan, 1999) with the following default parameters: (*d*  =  500, *g*  =  2, h =  100,000, *I*  =  30, *j*  =  31, *n*  =  −2, *s*  =  800, *t*  =  3000, *o*  =  16, *p*  =  80, *r*  =  0, *y*  =  50, *z*  =  5). The forward and reverse contigs that were supported by at least 10% and 60% reads, respectively, were merged using the Needleman–Wunsch global alignment algorithm (Needleman and Wunsch, 1970), in which the exact match achieves a score of 5. The merged contigs were considered as overlapped with the following conditions: the 10 headmost bases identical to the 10 headmost base in forward contig; the 10 backmost bases identical to the 10 backmost bases in reverse contig; the score larger than 50; the identity larger than 10; and the quotient of the score divided by the identity large than 4. The nonoverlapped forward contig and reverse contig were connected with 10 “N” as a separator.

The final contigs were investigated by comparing them with other reference genomes and counting SSR motifs. Four related fish genomes were selected for this purpose: three-spined stickleback (ASM18067v1) (Berner et al., 2019), Nile tilapia (Orenil1.1) (Brawand et al., 2014), European seabass (seabass_V1.0) (Tine et al., 2014), and zebrafish (GRCz10) (Howe et al., 2013). European seabass and yellow perch belong to the same suborder of Percoidei, and Nile tilapia and yellow perch belong to the same order of Perciformes, while three-spined stickleback and zebrafish belong to order of Gasterosteiformes and Cypriniformes, respectively. Blastn (Altschul et al., 1997) was used to align the sequences with *e* value 10^−6^ and of at least 100-bp alignment length.

### SNP and Indel Identification

The final contigs were connected with 100 “N” to be a pseudomolecule as the reference sequence. The original reads without trimming lengths were mapped to the reference sequence with the BWA-MEM software package version 0.7.15 (Li, 2013) with a limitation of a maximum insert size of 1 kbp, which was also used for polymorphic SSR detection below. SAMtools version 1.3.1 (Li et al., 2009) and Picard tools version 2.3.0 (http://picard.sourceforge.net) were used to manipulate the mapped files. Prior to calling markers, reads with an insert size over 1,000 bp were removed using an in-house Perl script, and properly paired reads were then selected using SAMtools (Li et al., 2009). Duplicated reads including optical duplications were filtered using Picard tools. Reads with Indels were realigned with RealignerTargetCreator and IndelRealigner of the GATK software package version 3.6 (McKenna et al., 2010). SNPs and Indels were called using SAMtools (Li et al., 2009) in a multiple-sample model and filtered using VCFtools version 0.1.15 (Danecek et al., 2011) with the following parameters: (1) base quality and map quality ≥20; (2) variant quality ≥300; (3) depth per sample ≥5-fold and <200-fold; (4) minor allele frequency >0.05. Three variant datasets were obtained with different and further filter. In the first dataset, the SNPs and Indels that existed in at least 10 individuals were selected. In the second dataset, 11 individuals were removed because of containing too few SNPs (**Figure 1E**). The remaining individuals were from six strains, each with three individuals (**Table 1**), and they could still represent the native population of this species. Those SNPs that existed in at least 80% of individuals and located from 11 to 80 bp on the forward contig were selected. In the third dataset, only one SNP from each forward contig in second dataset was selected.

### Population Genetics

The second dataset was used to estimate nucleotide diversity, including per-SNP nucleotide diversity (π_SNP_) and total nucleotide diversity (π_total_). Per-SNP nucleotide diversity was calculated using VCFtools version 0.1.15 (Danecek et al., 2011), and total nucleotide diversity was averaged across all the sites, including the invariant sites that meet the minimum depth in each individual and minimum percentage in all the 18 individuals (Lozier, 2014). Confidence interval for total nucleotide diversity was obtained by 10,000 bootstrap replicates across sites using package boot (Canty and Ripley, 2012) in R (Team, 2013).

The third dataset was used to infer the population structure. A phylogenetic tree by the maximum likelihood method was constructed using SNPhylo version 20140701 (Lee et al., 2014), and a principal component analysis was performed in R program LEA (Frichot and François, 2015), in which significance of the identified principal components was evaluated through Tracy–Widom statistics.

### Polymorphic SSRs Detection and Validation

Two programs were used to search for SSRs in the final contigs, Microsatellite search module (MISA, http://pgrc.ipk-gatersleben.de/misa/) and Tandem Repeats Finder version 4.07b (TRF) (Benson, 1999). MISA takes usage of regular expression pattern to scan the contigs from perfect SSRs, including di-, tri-, tetra-, penta-, and hexa-nucleotide motifs with numbers of uninterrupted repeat units more than 5, 4, 4, 4, and 4, respectively. TRF took usage of alignment score to recognize SSR with the following parameters: a match weight = 2; a mismatch and Indel penalty = 7; probability of a matching = 80%;probability of an Indel = 10%; maximum period = 500; the minimum scores for di-, tri-, tetra-, penta-, and hexa-nucleotide motifs = 22, 28, 28, 32, and 34, respectively (Benson, 1999; Willems et al., 2014).

Polymorphic SSRs were detected using the software lobSTR version 3.0.3 (Gymrek et al., 2012) according to the best practice. The SSR motifs were defined based on the result of TRF (Benson, 1999; Willems et al., 2014). The lobSTR genotyped SSRs with the following options: min-het-freq = 0.2, min-border = 5, min-bp-before-indel = 7, maximal-end-match = 15, min-read-end-match = 10, and max-matedist = 1000.

The raw genotyped SSRs with quality of at least 300, at least three alleles, and at least 200bp flanking sequence in each side were selected as high-quality SSRs. Primers were designed using Primer 3 (Andreas et al., 2012) in batch with SNPs and Indels being masked (the first dataset). The parameters were set as follows: (1) primer length ranging from 18 to 24 bases with optimal sizes of 21 nt; (2) PCR product size ranging from 125 to 250 bp; (3) melting temperature between 55°C and 65°C, with 60°C as the optimum annealing temperature; (4) a GC content of 40% to 60%, with an optimum of 50%. The premier pairs were *in silico* validated using re-PCR (Schuler, 1998) with parameters of two mismatches and two gaps, and those with only one production that existed were treated as high-quality primers. Forty pairs of primers were selected at random and then synthesized in Integrated DNA Technologies (Coralville, IA). The PCR reaction was conducted using Platinum^™ ^SuperFi^™^ Green PCR Master Mix (Invitrogen, Carlsbad, CA) and performed in a thermal cycler (Bio-Rad, Hercules, CA) under the following conditions: 30 s at 98°C; 35 cycles of 10 s at 98°C, 30 s at 55°C, 45 s at 72°C; and 5 min at 72°C. PCR products were visualized in a 2% agarose gel.

## Ethics Statement

All the methods and experimental protocols of this study were performed in accordance with guidelines and regulations approved by the animal ethics committee of The Ohio State University (USA) and the University of Wisconsin–Milwaukee (USA) Institutional Animal Care and Use Committee.

## Author Contributions

H-PW and BS conceived and designed the experiments. HY and OS-V performed the experiment. LG and D-CZ analyzed the data and prepared a draft of the manuscript. H-PW and BS revised and finalized the manuscript. All authors read and approved the manuscript.

## Funding

This work was financially supported by United States Department of Agriculture (No. 2010-38879-20946) and USDA-ARS CRIS project (5090-31320-003-00D). Salaries were provided by state and federal funds appropriated to The Ohio State University, Ohio Agricultural Research and Development Center.

## Conflict of Interest

The authors declare that the research was conducted in the absence of any commercial or financial relationships that could be construed as a potential conflict of interest.
